# The immune and metabolic changes with age in giant panda blood by combined transcriptome and DNA methylation analysis

**DOI:** 10.18632/aging.103990

**Published:** 2020-11-07

**Authors:** Xiaoyu Huang, Qingyuan Ouyang, Mingxia Ran, Bo Zeng, Linhua Deng, Shenqiang Hu, Mingyao Yang, Guo Li, Tao Deng, Ming He, Ti Li, Haidi Yang, Guiquan Zhang, Heming Zhang, Changjun Zeng, Jiwen Wang

**Affiliations:** 1 Farm Animal Genetic Resources Exploration and Innovation Key Laboratory of Sichuan Province, Sichuan Agricultural University, Chengdu 611130, Sichuan, China; 2 China Conservation and Research Center for the Giant Panda, Dujiangyan 611830, Sichuan, China; 3 Key Laboratory of State Forestry and Grassland Administration on Conservation Biology of Rare Animals in The Giant Panda National Park, Dujiangyan 611830, Sichuan, China

**Keywords:** giant panda, transcriptome, methylation, aging

## Abstract

Giant panda (*Ailuropoda melanoleuca*) is an endangered mammalian species. Exploring immune and metabolic changes that occur in giant pandas with age is important for their protection. In this study, we systematically investigated the physiological and biochemical indicators in blood, as well as the transcriptome, and methylation profiles of young, adult, and old giant pandas. The white blood cell (WBC), neutrophil (NEU) counts and hemoglobin (HGB) concentrations increased significantly with age (young to adult), and some indicators related to blood glucose and lipids also changed significantly with age. In the transcriptome analysis, differentially expressed genes (DEGs) were found in comparisons of the young and adult (257), adult and old (20), young and old (744) groups. Separation of the DEGs into eight profiles according to the expression trend using short time-series expression miner (STEM) software revealed that most DEGs were downregulated with age. Functional analysis showed that most DEGs were associated with disease and that these DEGs were also associated with the immune system and metabolism. Furthermore, gene methylation in giant pandas decreased globally with age, and the expression of *CCNE1*, *CD79A*, *IL1R1*, and *TCF7* showed a highly negative correlation with their degree of methylation. These results indicate that the giant panda’s immune function improves gradually with age (young to adult), and that changes in the methylation profile are involved in the effects of age on immune and metabolic functions. These results have important implications for the understanding and conservation of giant pandas.

## INTRODUCTION

The giant panda (*Ailuropoda melanoleuca*) is a first-class national protected species in China. Over the past 20 years, extensive research on the protection of giant pandas has been conducted all over the world. Previous studies showed very little difference in the physiological and biochemical indexes of giant panda blood between males and females, while several indicators, including the number of immune cells, were found to be linearly related to age [1]. The immune system undergoes profound transformations with age, and these changes are globally known as ‘immunosenescence’ in mammals [2]. Immune cells and circulating factors, including chemokines, cytokines, and other soluble molecules, are generally considered to be critical factors that affect the immune system during the aging process [3]. Exploring the changes in the giant panda’s immune cells with age and the underlying mechanisms is essential to developing strategies that delay or prevent the age-related decline in immunity. Aging is arguably the universal contributor to the etiologies of metabolic decay and related diseases, including type 2 diabetes mellitus, cardiovascular disease, and insulin resistance [4, 5]. Substantial evidence suggests that aging organisms exhibit homeostasis disorders of carbohydrate, amino acid, and fatty acid metabolism [6, 7]. Enhancing mitochondrial function or inhibiting glycolysis can extend lifespan and promote healthy aging in many species [8]. However, research on age-related changes in immune and metabolic functions of giant pandas is still lacking.

Two recent transcriptomic studies [9, 10] of the changes in gene expression with age in young and old giant pandas showed that the differentially expressed genes (DEGs) identified in the blood were associated with immunity. Nevertheless, aging involves two processes (young to adult and adult to old), and separate studies of these two processes are still lacking. Furthermore, changes in gene expression may be caused by DNA methylation [11], a process that has been widely reported to be involved in the aging process in humans and mouse [12, 13]. Approximately 0.5% of the genome in pandas is methylated [14] and DNA methylation has been shown to be involved in the regulation of cataract disease in old giant pandas [15]. However, the molecular mechanism of age-related immune and metabolic changes in giant pandas has not yet been systematically investigated using multi-omics technology.

In this study, we systematically analyzed the physiological and biochemical changes in the blood of giant pandas with age and investigated the underlying molecular mechanisms. We identified age-related DEGs and used the short time-series expression miner (STEM) to classify the age-related DEGs (upregulated and downregulated). Each type of DEG was used for functional enrichment analysis, and the expression and degree of methylation of the functional DEGs were used for correlation analysis. Furthermore, we constructed the first age-related dynamic methylation profile of panda blood. Identification and functional analysis of differentially methylated genes (DMGs) was also conducted. This research is of great significance as the basis of further investigations aimed at developing strategies for the protection of the giant panda.

## RESULTS

### Physiological and biochemical indicators in blood change with age

**Figure 1:**
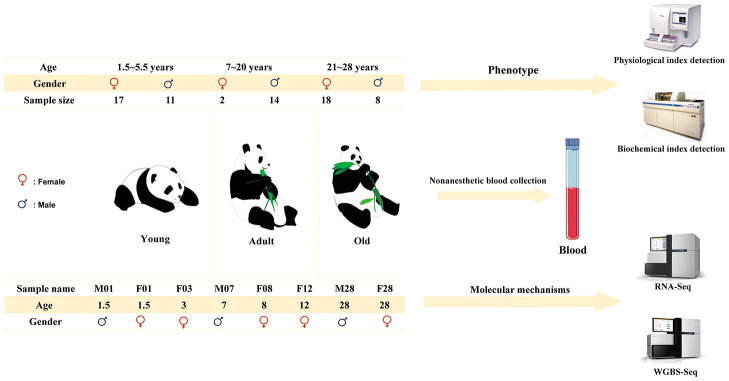
Schematic diagram of experimental process design.

As shown in Figure 1, the physiological and biochemical indexes in the blood of the giant pandas of different ages were systematically determined and analyzed (Supplementary Table 1). The white blood cell (WBC) and neutrophil (NEU) counts and hemoglobin (HGB) concentrations increased significantly (*P* < 0.05) young to adult, while decrease significantly from adult to old (Table 1). Furthermore, the levels of chloride ions (Cl^-^) in the blood decreased significantly with age (*P* < 0.05), while the levels of glutamate transaminase (AST) and glutamyl transpeptidase (GGT) increased significantly (*P* < 0.05). Some physiological and biochemical indicators related to metabolism in blood were also found to change significantly with age. Blood glucose (GLU) levels decreased with age, while triglycerides (TG) and apolipoprotein B (APOB) levels increased (Table 1). Furthermore, cholesterol (CHOL) levels were significantly higher in old giant pandas compared with those in adult giant pandas (*P* < 0.05).

**Table 1:** Blood physiological and biochemical indicators closely related to age.

**Project**	**Young**	**Adult**	**Old**	***P***	**Young *vs.* Adult**	**Young** *vs.* Old	**Adult** *vs.* Old
WBC (10^9^/L)	6.14±0.21	7.55±0.39	6.04±0.32	0.002^**^	0.002^**^	0.797	0.001^**^
NEU (10^9^/L)	4.34±0.21	5.71±0.34	4.84±0.23	0.002^**^	0.000^**^	0.131	0.024^*^
NEUT (%)	66.55±2.84	75.59±1.93	78.06±0.83	0.000^**^	0.007^**^	0.000^**^	0.456
HGB (U/L)	118.29±1.52	125.44±3.77	115.04±2.00	0.013^*^	0.037^*^	0.274	0.003^**^
Na^+^ (mmol/L)	126.41±0.44	125.85±0.68	124.80±0.51	0.068	0.480	0.022^*^	0.195
Cl^-^ (mmol/L)	96.09±0.44	94.96±0.70	93.25±0.48	0.000^**^	0.151	0.000^**^	0.035^*^
AST (mmol/L)	56.43±1.97	63.31±2.63	70.65±2.54	0.000^**^	0.059	0.000^**^	0.047^*^
GGT (mmol/L)	6.57±0.27	5.75±0.51	10.65±1.12	0.000^**^	0.487	0.000^**^	0.000^**^
GLU (mmol/L)	4.671±0.12	4.581±0.27	4.01±0.14	0.006**	0.707	0.002**	0.022*
TG (mmol/L)	1.544±0.092	1.911±0.097	2.098±0.097	0.000^**^	0.015*	0.000^**^	0.215
CHOL (mmol/L)	5.594±0.183	4.841±0.28	5.728±0.358	0.115	0.086	0.723	0.047^*^
HDL (mmol/L)	3.769±0.076	2.945±0.111	3.019±0.096	0.000^**^	0.000^**^	0.000^**^	0.602
LDL (mmol/L)	3.312±0.137	2.846±0.223	3.463±0.258	0.161	0.149	0.586	0.060
APOA1 (g/L)	0.696±0.021	0.632±0.03	0.66±0.022	0.187	0.077	0.245	0.447
APOB (g/L)	0.02±0.002	0.032±0.005	0.033±0.003	0.008^**^	0.019^*^	0.004^**^	0.867

Note: Most commonly used hematologic and biochemical parameters were tested. The marked * was a significant difference (*P<0. 05*), and the marked ** was an extremely significant difference (*P<0.01*). The unmarked letter indicated no statistical difference.

### Identify the DEGs

**Figure 2:**
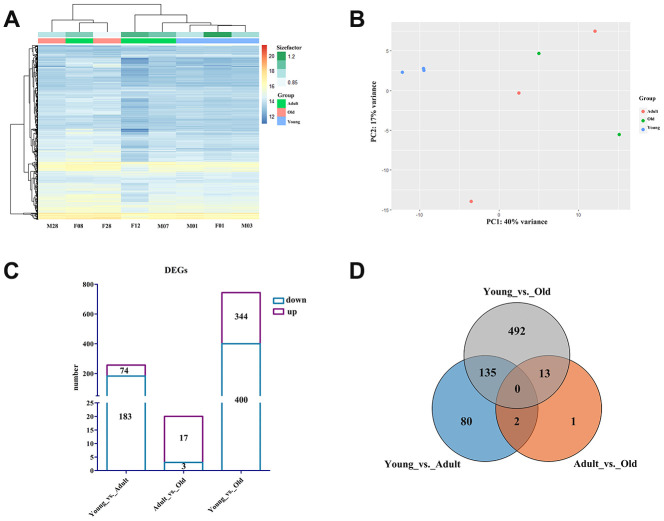
**Profile of the giant panda blood transcriptome.** (**A**) Heat map plot of all genes using TPM expression value of genes by adopting hierarchical clustering method. (**B**) PCA of all genes using TPM expression value of genes by adopting hierarchical clustering method. (**C**) The histogram of the number of DEGs in each group. The purple box represents the number of upregulated DEGs, and the blue box represents the number of downregulated DEGs. (**D**) Venn diagram of the number of annotated DEGs in each group.

To identify of the DEGs between different ages, we constructed cDNA libraries derived from blood samples to obtain the normalized expression of each gene, using |log2FoldChange| > 1 and false discovery rate (FDR) <0.05 as the standard for screening DEGs. Eight cDNA libraries were constructed using total RNA from blood samples obtained from giant pandas, and a total of 657,404,608 reads were obtained. Reads from the samples covered an average of 89.80% (88.45%–90.86%) of the giant panda reference genome [16], and an average of 86.33% (84.0%–87.8%) of the reads were compared only once (Supplementary Table 2). We used the expression levels of all genes for principal component analysis (PCA) and clustering heat map analysis. As shown in Figure 2A, all samples from the young group and two samples from the adult group were clustered together, and one sample from the adult group and all samples from the old group were clustered into a separate cluster. PCA results showed that all samples in the young group were separated from the samples in the other groups, and the samples in the adult and old groups could not be distinguished effectively (Figure 2B). In total, we detected 257, 20, and 744 DEGs in the Young vs. Adult, Adult vs. Old, and Young vs. Old groups (Figure 2C). The Young vs. Adult and Young vs. Old groups had the highest proportion of overlapping DEGs, while the Young vs. Old group has the most unique DEGs (Figure 2D).

### Functional analysis of DEGs

**Figure 3:**
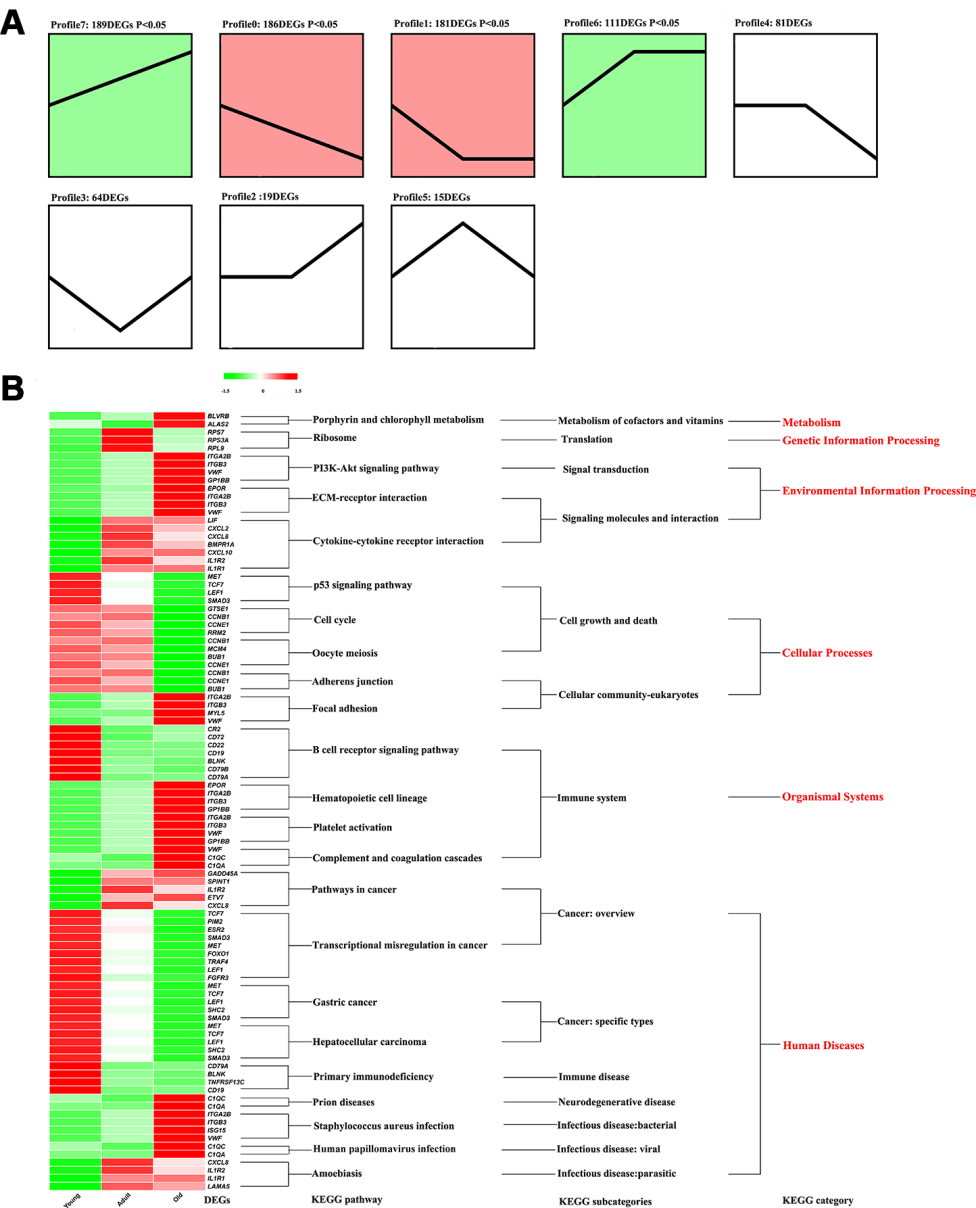
**The functional analysis of DEGs.** (**A**) Identify different profile through STEM. Trend blocks with color: Trends with significant enrichment, trend blocks with similar trends have the same color. Trend blocks without color: Trends with no significant enrichment. (**B**) KEGG pathways significantly enriched by DEGs in each profile.

Clarification of the function of DEGs is of considerable significance in understanding the physiological changes that occur with age in giant pandas. The protein interaction network (PPI) constructed based on the DEGs showed that *BUB1* (nodes = 26) and *CCNB1* (nodes = 26) had the most nodes (Supplementary Figure 1). As shown in Figure 3A, all the DEGs were divided into eight profiles by STEM according to the transcripts per million (TPM) in the three stages. However, only Profile7, Profile0, Profile1, and Profile6 showed significantly enrichment (*P* < 0.05). Profile7 contained the largest number of DEGs; however, due to the scattered functions of the DEGs, no significant KEGG pathway was found to be enriched in Profile7 (Supplementary Table 3). As shown in Figure 3B, KEGG pathways associated with metabolism, genetic information processing, environmental information processing, cellular processes, organismal systems, and human disease categories were enriched by the DEGs in the different profiles. To be more specific, the porphyrin and chlorophyll metabolism pathways belonging to the metabolism category were significantly upregulated in old giant pandas. All the DEGs enriched in the environmental information category pathways showed the lowest expression in the young giant pandas, and the DEGs enriched in the PI3K-Akt signaling and ECM-receptor interaction pathways showed the highest expression in the old giant pandas. DEGs enriched in most pathways of the cellular processes category showed the highest expression in the old giant pandas, while the expression levels of DEGs in the focal adhesion pathway increased with age. All pathways in the category of organismal systems belonged to the immune system subcategories. The DEGs in the B cell receptor signaling pathway showed the highest expression levels in the young pandas, and the other immune system-related pathways were found to be activated in the old giant pandas. The largest number of KEGG pathways belonged to the human disease category, although the expression trends varied among the different disease pathways.

### Analysis of DNA methylation patterns

We constructed the first age-related dynamic methylation map of the giant panda with age and obtained a total of 830.13 Gb of original data (Supplementary Table 4). Comparison of the methylation sites in each sample revealed that the cytosine (C) methylation rate of the eight genomic DNA samples was approximately 5%, with the methylation rate at the CG site, while methylation at the C sites of CHG and CHH accounted for a smallest proportion among the C sites in the genome (Supplementary Figure 2A). In addition, the ratio of methylated CG:CG in all methylated cytosine sites and the ratio of CG-methylated cytosine in all methylated cytosine sites ranged from 78.21%–81.01%, while less than 1% of methylated cytosines were mCHG and mCHH, indicating that methylation of the giant panda genome mainly occurs at CG sites (Supplementary Table 5). The levels of cytosine methylation observed in each functional element in the eight samples were similar in three sequence environments (Supplementary Figure 2B). DNA methylation levels in introns and the 3'-untranslated region (3'-UTR) were higher in the CG sequence environment, followed by the CGI shore, repeat, and exon regions, while DNA methylation levels were lowest in promoter regions. Furthermore, in the promoter region, the proximal region had the lowest DNA methylation level, while the distal region had the highest DNA methylation level. In the CHG and CHH sequences, the level of DNA methylation in the CGI region was the highest, followed by the CGI region and the 5'-UTR region, and the methylation levels of the remaining functional elements were almost unchanged. We also analyzed the density of methylation in the gene coding region and the regions 2 kilobases (2 Kb) upstream and downstream of the gene (Supplementary Figure 2C). In the CG sequence environment, all samples showed the highest methylation levels in the region 2 K downstream of the gene, and the lowest in the region 2 K upstream. In the mCHG/mCHG sequence environment, we found that most individuals had a methylation peak in the region 2 K downstream of the gene. In contrast, the methylation levels in the other regions remained unchanged and were maintained at a low level. In the mCHH/mCHH environment, the methylation level was also high in the region 2 K downstream of the gene and remained low in the other regions.

### Identification and functional analysis of DMGs

The differently methylated regions (DMRs) in each group were identified using the ‘methylKit’ package. The number of DMRs in the Young vs. Adult, Adult vs. Old, and Young vs. Old groups were 4,273, 6,544, and 8,834, respectively. In addition, the number of DMGs in the Young vs. Adult, Adult vs. Old, and Young vs. Old groups were 466, 823, and 991, respectively. In all groups, there were greater numbers of hypomethylated than hypermethylated DMRs and DMGs. The proportions of demethylated DMGs in each group were 82.5%, 93.0% and 94.5%, respectively, suggesting that methylation patterns decrease globally with age. The KEGG pathways significantly enriched by DMGs in each group are shown in Supplementary Table 6. The KEGG pathways enriched in the three groups included pathways belonging to the categories of environmental information processing, organismal systems, and human diseases. There were also pathways belonging to cellular process in the Adult vs. Old group.

### Combined analysis of transcriptome and Whole Genome Bisulfite Sequencing (WGBS) data

**Figure 4:**
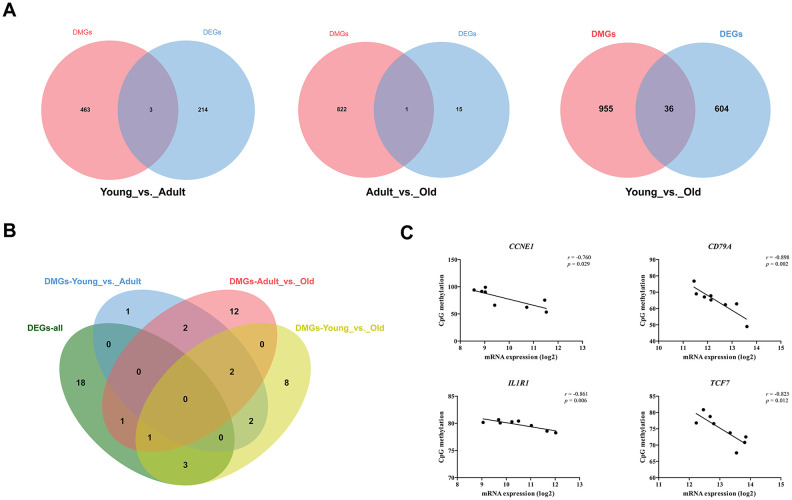
**Combined analysis of transcriptome and methylation data.** (**A**) Venn diagram of DEGs and DMGs in different groups. (**B**) Venn diagram of KEGG pathway significantly enriched by DEGs and DMGs. (**C**) A scatter plot and trend line (Pearson correlation) showing correlation between the log2 ratios of *CCNE1*, *CD79A*, *IL1R1*, and *TCF7* expression from transcriptome and CpG methylation.

To determine whether DNA methylation is involved in the regulation of DEG expression, we first identified the intersection of DEGs and DMGs in each age group. As shown in Figure 4A, there were three, one, and 36 genes that were both differentially methylated and differentially expressed in the Young vs. Adult, Adult vs. Old, and Young vs. Old groups, respectively (Supplementary Table 7). Furthermore, we found that the KEGG pathways enriched by DMGs in each group overlapped with the KEGG pathways enriched by DEGs in all profiles (Figure 4B). In addition, 49 DEGs that were significantly enriched in the KEGG pathways were selected from each profile based on previous research. We obtained the expression levels, CpG methylation values in the promoter region and CpG methylation values for each of these DEGs. Then, we analyzed potential correlations between the expression and CpG methylation levels of the 49 DEGs. The expression levels of *CCNE1, CD79A1, IL1R1*, and *TCF7* were highly correlated with the degree of CpG methylation. The expression of *CCNE1* was negatively correlated with CpG methylation in the promoter region (r = -0.760). The expression of *CD79A, IL1R1,* and *TCF7* was negatively correlated with CpG methylation in the gene body region. In the PPI network constructed for all DEGs, *CCNE1, CD79A, IL1R1,* and *TCF7* all had more than five nodes, suggesting that these genes play an important role in the age-related changes in the entire genetic network.

## DISCUSSION

### The immune-related indicators of giant pandas changed with age

Most of the immune-related physiological indicators in giant pandas indicated that immune function improved gradually from the young to the adult age group. WBCs, including NEU, monocytes (MONO), eosinophils (EOS), basophils (BAS), and lymphocytes (LY), are components of the innate and adaptive immune systems [17]. HGB is an iron-associated protein that is required to for oxygen transport in mammals [18]. Activation of the immune system leads to increased expression of transferrin receptors on NEUs, decreased serum iron levels (transferrin), and increased intracellular iron due to deposition by ferritin. T cell-dependent immune responses are reduced as a result of iron deficiency; thus, our findings suggest that HGB is also involved in the humoral immune response [19]. In our results, the number of WBC, NEU and HGB increased significantly from childhood to adulthood, but decreased significantly from adult to old age. This shows that the immune function of giant pandas improved gradually from young to adult, while decreased gradually from adult to old. Inflammation plays a crucial role in the control of pathogens and the formation of subsequent adaptive immune responses [20]. AST and GGT significantly increased as the pandas aged from adult to old, suggesting that the occurrence of inflammatory reaction gradually increased during the aging process [21]. This observation was supported by the changes in Cl^-^ and Na^+^ concentrations. Cl^-^ are the main ions in valval cells and are usually accompanied by Na^+^. Our results showed that the Cl^-^ and Na^+^ concentrations decreased with age. The reduction of Cl^-^ is thought to enhance the inflammatory response of endothelial cells [22].

### Unique changes in metabolism-related indicators during the aging process in giant pandas

Intriguingly, aging is often accompanied by whole-body dysregulation of cholesterol metabolism [23]. A clinical manifestation of this process is an increase of low density lipoprotein cholesterol (LDL) and a decrease of high density lipoprotein (HDL) with age [24]. Compared with adult giant pandas in our study, plasma CHOL and LDL levels were obviously increased in old giant pandas. However, HDL levels increased slightly in the plasma of old giant pandas, which is in contrast to the results of studies in other mammals [24]. HDL is transported to the liver and then removed through the intestine and this is the mechanism of excess cholesterol removal in peripheral tissues; therefore, HDL levels are closely related to lifespan [25, 26]. Normally, HDL levels decreases by 1% per year, although favorable HDL characteristics are often observed in the offspring of centenarians [27], indicating that increased HDL levels confer exceptional longevity. In addition to the differences in HDL levels compared with the aging characteristics of other mammals, our results show that the GLU levels of giant pandas are also inconsistent with most mammalian aging characteristics. In aged mammals, the basal rate of gluconeogenesis is increased leading to increased blood glucose levels, while the hepatic association of glucose with glycogen is reduced [28]. In contrast to gluconeogenesis, the absorption of glucose by skeletal muscle, brain, and other energy-consuming tissues decreases with age, due to reduced insulin signaling [29], reduced insulin sensitivity [30], and reduced levels of glucose transporter [31]. This imbalance in supply and demand often leads to the occurrence of disorders of glucose metabolism in old age. The GLU levels of giant pandas in this study decreased significantly in old age, which is completely opposite to the pattern observed in other mammals. Multiple studies have shown that diet can reduce the effects of aging on cholesterol and glucose metabolism [32, 33]. Therefore, we speculate that the giant panda’s diet may be one of the reasons that its metabolic ability changes with age in a manner that is different from other mammals.

### The susceptibility of giant pandas to different diseases changes with age

In order to further explore the molecular mechanism behind this change in blood physiological and biochemical indicators, we performed transcriptome sequencing on the blood of giant pandas of different age groups. However, the gene expression patterns of the three samples in the adult group are not completely similar, and the expression pattern of F08 is more similar to that of the two 28-year-old giant pandas. We are not clear what mechanism caused the specific expression pattern of F08, but such results suggest that there are differences in the expression patterns of different individuals at the same age. This may also be one of the reasons why there are fewer DEGs between the blood transcriptomes of adult and old giant pandas. Moreover, we used STEM software to divide the expression patterns of all age-related DEGs. In the KEGG pathway enriched by DEGs with different expression trends, most of belonged to the category of human diseases, indicating age-related variation in the risk of pandas suffering from diseases. Du et al.'s report [10] also showed that 27 of the 35 KEGG pathways enriched in DEGs between adult and old giant pandas are disease-related. In the human disease category, most pathways were enriched in cancer-related subcategories. In addition to the cancer pathway, the expression of DEGs in pathways in other cancer subcategories decreased continuously with age. And some recent research reports have shown the existence of ovarian cancer and testicular cancer in old giant pandas [34]. These results suggest that the risk of cancer in giant panda changes with age. In addition, the expression levels of DEGs enriched in the primary immunodeficiency pathway decreased significantly from young to adult and then stabilized, indicating that the innate immune system of giant pandas gradually improved with development from the young to the adult stage. The expression levels of DEGs associated with infection-related pathways were lowest in the young and usually peaked in old age. Unlike diseases such as cancer, infectious diseases are an important threat to the giant panda population [35, 36]. These results suggest that the prevention of infectious diseases may be important in extending its the lifespan of giant pandas in old age.

### Transcriptome analysis indicates the immune and metabolic functions of giant pandas change with age

In accordance with the changes in blood physiological and biochemical indicators, DEGs with different trends were also significantly enriched in the pathways related to immune system and metabolism. Among the immune-related pathways, DEGs other than the B cell receptor signaling pathway showed the highest expression levels in old giant pandas. Multiple genes in the CD family, including *CD19, CD22, CD72, CD79A,* and *CD79B*, were enriched in B cell receptor signaling pathway. *CD79A* and *CD19* were reported to inhibit the conversion of pro-B cells to pre-B cells [37]. *CD19* [38] and *CD22* [39] inhibit B cell proliferation under certain conditions, with significant downregulation of their expression in adults suggesting that this this inhibitory effect is weakened. These DEGs (*CD79A, CD19, CD22*) have a negative regulatory effect on B cell maturation, and their upregulation at an early age also indicates that the immune system of the young giant panda is not yet fully mature. Hematopoietic stem cells (HSC) refer to cells that have not yet matured and are the origin of all hematopoietic cells and immune cells. HSC can differentiate into red blood cells, WBC, and platelets. The function of HSC deteriorates with age with a concomitant effect on the function of the regenerative hematopoietic system [40]. However, in this study, hematopoietic cell profiles, platelet activation, complement and coagulation cascade pathways were all upregulated in old age. Blood physiological and biochemical indicator analyses also showed that the numbers of red blood cells and platelets were slightly reduced in the old giant pandas (*P* > 0.05). Collectively, these results suggest that there may be a mechanism that protects the function against the aging of HSC in pandas. Interestingly, we also found that the porphyrin and chlorophyll metabolism pathways were significantly upregulated in the old giant pandas. Chlorophyll plays role in reducing plasma levels of GLU [41] and CHOL [42], which may explain the difference in the trend of GLU and CHOL indicators between giant pandas and other mammals with age.

### DNA methylation is involved in the changes of immune and metabolic functions of giant pandas with age

To further explore the regulatory mechanisms underlying these changes in immune and metabolic functions, we constructed the first dynamic map of blood DNA methylation in the giant panda. Previous studies have shown that CG methylation is the major type of methylation in mammals, including humans [43], mice [44], and poultry [45]. In accordance with this, we showed that the ratio of all methylated C sites and mCG in all methylated cytosine sites was 78.21%–81.01%, while less than 1% of the methylated cytosines were mCHG and mCHH. DNA methylation decreases globally with age. These results are similar to those found in other mammals [12, 46], indicating the DNA methylation patterns of mammals are similar and conserved. DNA methylation is thought to be involved in the regulation of gene expression, usually by inhibition [47]. However, only few of DEGs in each group are also differently methylated, this may be due to the fact that gene expression is also regulated in many ways other than methylation [48, 49]. In this study, we showed overlaps between the DEGs and DMGs in each group as well in the KEGG pathways enriched by DEGs and DMGs. *CCNE1, CD79A, IL1R1,* and *TCF7* were identified as DEGs with multiple nodes in the PPI, and their methylation and expression showed a significant negative correlation. *CCNE1* as a gene related to proliferation is increased in normal aging and carcinogenic people [50]. *CD79A* plays an important role in immunity. Studies in humans have shown that *ILI1R1* increases with age [51], and its increase is associated with high blood pressure, diabetes, sudden heart failure, and a higher risk of mortality [52]. In particular, the age-related inhibition of *TCF7* expression by methylation not only plays an important role in immune enhancement [53] and tumor suppression [54], but also in balancing blood glucose levels [55] and hematopoiesis [56]. More importantly, as a transcription factor, the methylation of *TCF7* may affect the expression levels of other functional genes. These results indicate that DNA methylation is involved the process by which biological functions alter with age in the giant panda.

In general, our experimental system explains the age-related changes in blood physiological and biochemical indicators, transcriptome and methylation group map in giant pandas. Such results indicate that giant pandas have unique physiological characteristics at each age stage, so we need to differentiate the breeding of giant pandas of different ages according to their physiological characteristics. Moreover, Our results indicate that gene methylation participates in changes in immune and metabolic functions with age by regulating gene expression. These results provide an important reference in the development of strategies for the protection of giant pandas.

## MATERIALS AND METHODS

### Ethics statement

All experiments involving animals in this study were conducted in strict accordance with the animal protection laws of the People’s Republic of China (October 26, 2018). Protocols for animal studies were approved by the Care and Use of Laboratory Animals of the Animal Ethics Committee of China Conservation and Research Center for the Giant Panda (Dujiangyan, China) (Approval No. 20180212) and the Key Laboratory of State Forestry and Grassland Administration on Conservation Biology of Rare Animals in the Giant Panda National Park.

### Detection of physiological and biochemical indexes in blood

Thirty captive giant pandas aged 1–28 years were raised in China Conservation and Research Center for Giant Panda (Dujiangyan, China). These pandas were healthy (except for a few giant pandas with medical history) with typical spirit, appetite, and defecation patterns (Supplementary Table 8). During the period between 2017 and 2019, and 70 blood samples were collected as follows: 28 from the young group (1.5–5.5 years old), 16 from the adult group (7–20 years old), and 26 from the old group (21–28 years old). All samples were sent to Dujiangyan People’s Hospital for physiological and biochemical tests using the bc-5800 hematology analyzer (Mindray Technology Co., Ltd., China) and AU 2700 chemistry analyzer (Olympus, Japan), respectively.

### Sample collection, library preparation, and sequencing

Blood (10 mL) were sampling from giant pandas in the young (n = 3), adult (n = 3), and old (n = 2) groups (Table 2). Total RNA and DNA were extracted from the blood samples using TRIzol Reagent and Blood tissue DNA extraction (Qiagen, Hilden, Germany), respectively. Qualified RNA and DNA were sent to Novogene (Tianjin, China) where libraries were generated. Subsequently, transcriptome and genome-wide methylation sequencing was conducted using the Illumina HiSeq 2000 and Illumina HiSeq 2500, respectively. All sequencing data have been submitted to the NCBI database (https://dataview.ncbi.nlm.nih.gov/object/PRJNA615061?reviewer=g4s40ik50kbdmmmfjfg3obtdnf).

**Table 2:** Information on sequencing samples used in our study.

**Sample name**	**Age (year)**	**Gender**	**Genetic background**	**Group**
F01	1.5	Female	F4 of A × F1 of B	Young
M01	1.5	Male	F3 of A × F1 of B	Young
M03	3	Male	F3 of A × F2 of B	Young
M07	7	Male	F1 of B × C	Adult
F08	8	Female	F2 of A × D	Adult
F12	12	Female	F2 of A × E	Adult
M28	28	Male	Wild	Old
F28	28	Female	A × F	Old

### RNA-seq data analysis

Clean reads were compared to the giant panda reference genome (GCF_000004335.2) using HISAT2 (version 2.1.0). The output SAM (sequencing alignment/mapping) file was converted to a BAM (binary alignment/mapping) file and sorted using SAMtools (0.1.19-44428cd). FeatureCounts (version 2.0.0) was used obtain the number of counts per gene. We divided the samples into Young *vs.* Adult, Young *vs.* Old and Adult *vs.* Old groups, and wrote a grouping file for each group. DEseq2 in R studio was used to read the data and group. After determination of the different genes in each group, the files were screened according to the FDR values. The DEGs were filtered based on FDR<0.05 and |log2FC|>1. String (https://string-db.org/) was used to construct a protein-protein interaction network for all the DEGs. The DEGs in each group were combined and their expression levels were input into STEM [57] software for analysis. A fold-change in expression >1.2 was considered to be a trend, and the genes were eventually divided into eight profiles. The KEGG functions of the DEGs in each profile were analyzed in KOBAS3.0 (http://kobas.cbi.pku.edu.cn/).

### BS-seq data analysis

Clean reads were deduplicated and aligned against the bisulfite-converted reference sequence using Bismark Bisulfite Mapper (v0.15.0). Methylation status was determined using the Bismark Methylation Extractor Script and only uniquely aligned reads, the number of methylated and unmethylated CpG, and non-CpG (CHG and CHH, H representing A/C/T) sites were counted for each individual cytosine. ‘MethylKit’ in the R package was used to identify the differently methylated CpGs (DMCs) and DMRs. Genes that overlapped with DMR were defined as DMGs. DAVID software (https://david.ncifcrf.gov/) was used to analyze the function of DMGs, and the results were considered significant when *P* < 0.05. Genome-wide DNA methylation analysis was conducted according to the annotated protein-coding genes in giant panda, which was downloaded from the Ensembl (http://asia.ensembl.org/index.html) database. The region from the transcription start site (TSS) to the transcription termination site (TTS) was defined as the gene body region. The genomic region 2.2 kb upstream of the TSS to 500 bp downstream of the TSS was considered as the proximal promoter region. CGI shores were defined as regions 2 kb in length adjacent to CGIs.

### Combined analysis of transcriptome and WGBS data

The correlation coefficient and *P-*value between the log_2_ the value of Transcripts per million reads (TPM) and methylation of the gene body and promoter regions were calculated using Hmisc (https://cran.r-project.org/web/packages/Hmisc/) in the R package.

## Supplementary Material

Supplementary Figures

Supplementary Tables 1-5

Supplementary Table 6

Supplementary Table 7

Supplementary Table 8
