# Supplementary material for: The immune and metabolic changes with age in giant panda blood by combined transcriptome and DNA methylation analysis
Source: Aging (Albany NY). 2020 Nov 7;12(21):21777–97. doi: 10.18632/aging.103990 (PMC11623972; doi:10.18632/aging.103990)
Supplement: Supplementary Figures [file aging-12-103990-s001.pdf]

SUPPLEMENTARY FIGURES

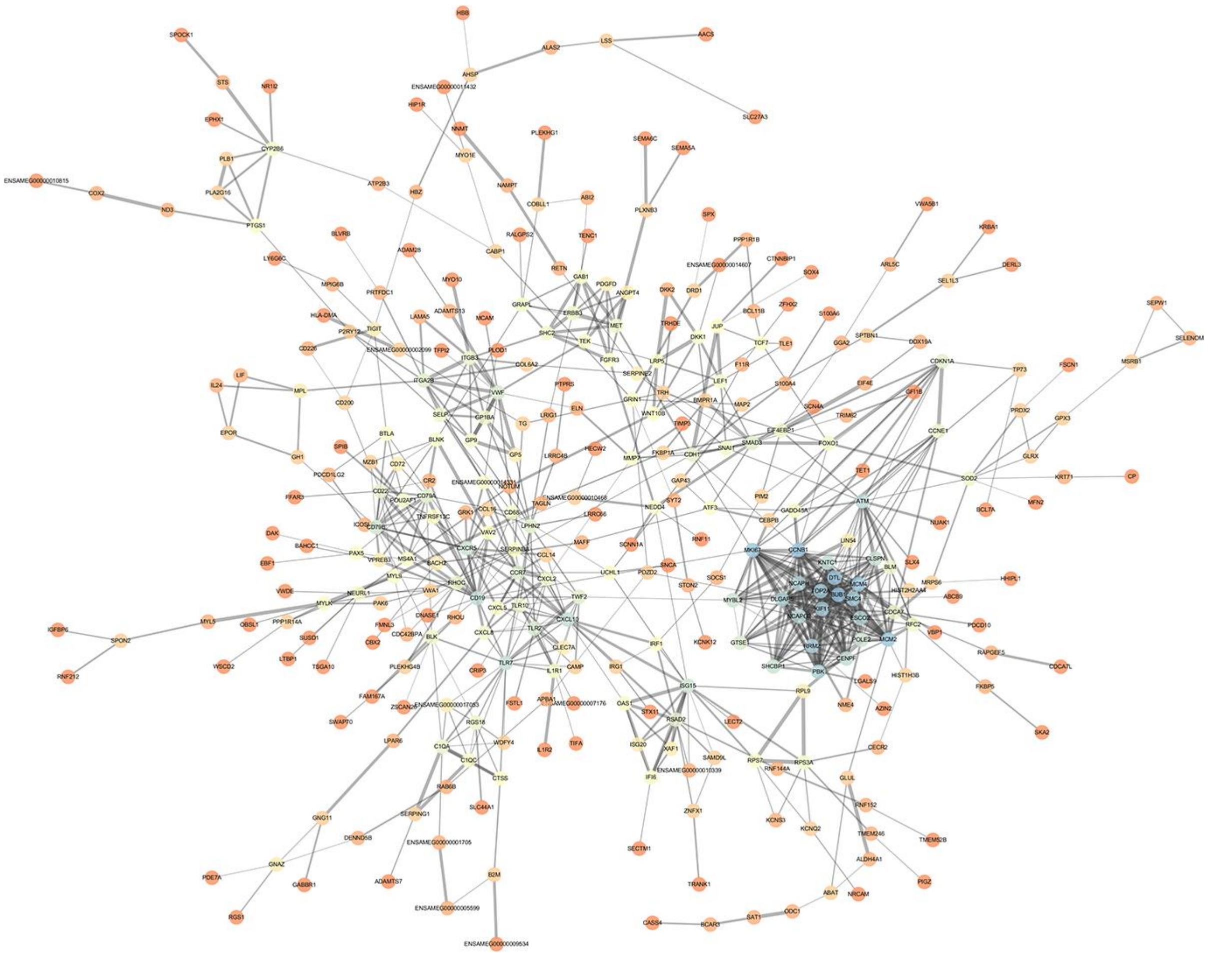

Supplementary Figure 1. Protein interaction network of all the DEGs. The thickness of the line indicates the combine score between the two proteins, and the thicker the line indicates the larger the combine score. Number of nodes heat map marks: orange to blue indicates number of nodes from low to high.

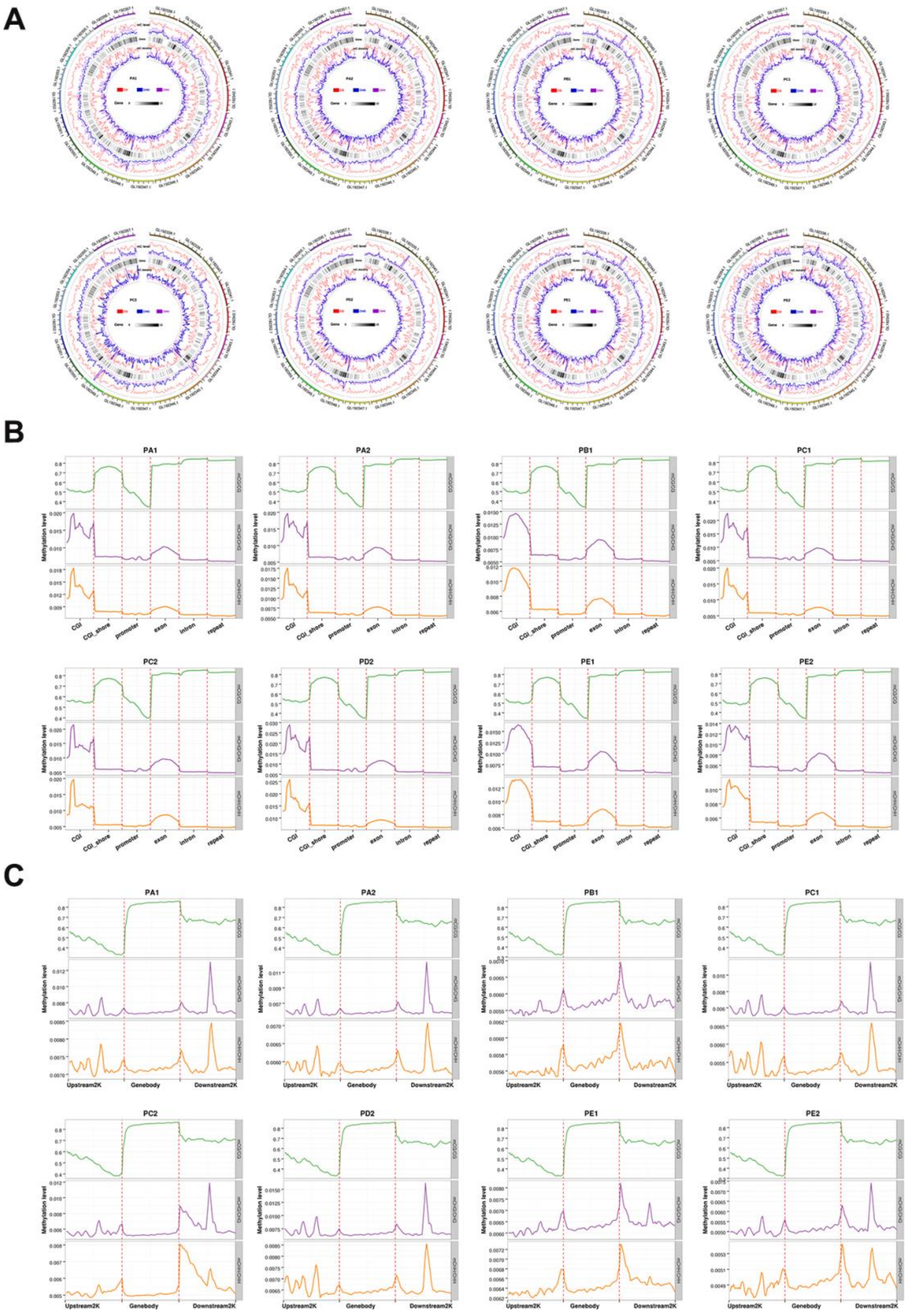

Supplementary Figure 2. Analysis of the methylated level of each sample. (**A**) Circos of chromosome methylation level. From outside to inside: CG sequence environment methylation density, CHG sequence environment methylation density, CHH sequence environment methylation density, TE original proportion of density heat map, gene number density heat map; internal scale: MC density Thermal standard: Green to yellow to red indicates methylation density from low to high, TE ratio Thermal standard: Green to black to red indicates repeat sequence ratio from low to high, gene density thermal standard: from gray to black Indicates that the number of genes is from low to high. (**B**) Distribution of methylation levels in samples on different genomic components. The abscissa represents the different genome elements, and the ordinate represents the methylation level. The functional regions of each gene were divided into 20 bin, and then the C locus level of the corresponding functional regions of all genes was averaged. Different colors represented different sequence contexts (CpG, CHG, CHH). (**C**) Distribution of sample methylation level in 2K upstream and downstream of the gene body. The abscissa represents the different regions, and the ordinate represents the methylation level. Each region of each gene was equally divided into 50 bin, and then the C locus level of the corresponding bin of all regions was averaged. Different colors represented different sequence contexts (CpG, CHG, CHH).
