# Supplementary material for: The immune and metabolic changes with age in giant panda blood by combined transcriptome and DNA methylation analysis
Source: Aging (Albany NY). 2020 Nov 7;12(21):21777–97. doi: 10.18632/aging.103990 (PMC11623972; doi:10.18632/aging.103990)
Supplement: Supplementary Tables 1-5 [file aging-12-103990-s002.pdf]

SUPPLEMENTARY TABLES

Supplementary Table 1. Blood physiological and biochemical indicators in young, adult, and old groups.

Project	Young group	Adult group	Old group	*P*	Young *vs* Adult	Young *vs* Old	Adult *vs* Old
WBC (10^9/L)	6.135±0.21	7.552±0.39	6.035±0.32	0.002**	0.002**	0.797	0.001**
Neu% (%)	66.55±2.84	75.594±1.93	78.058±0.83	0.000**	0.007**	0.000**	0.456
Lym% (%)	25.692±0.95	17.381±1.09	17.642±0.78	0.000**	0.000**	0.000**	0.854
Mon% (%)	3.362±0.3	3.275±0.43	2.942±0.38	0.666	0.873	0.386	0.545
Bas% (%)	0.323±0.047	0.213±0.043	0.192±0.046	0.090	0.119	0.039*	0.771
Neu (10^9/L)	4.338±0.207	5.711±0.34	4.841±0.231	0.002**	0.000**	0.131	0.024*
Lym (10^9/L)	1.539±0.061	1.265±0.089	1.089±0.068	0.000**	0.011*	0.000**	0.103
Mon (10^9/L)	0.158±0.02	0.174±0.041	0.124±0.022	0.404	0.686	0.323	0.207
Bas (10E9/L)	0.017±0.002	0.014±0.003	0.006±0.001	0.001**	0.501	0.000**	0.011*
RBC (g/L)	5.926±0.075	6.235±0.161	5.962±0.111	0.150	0.063	0.803	0.107
HGB (g/L)	118.286±1.52	125.438±3.773	115.04±1.996	0.013*	0.037*	0.274	0.003**
HCT (%)	30.882±0.404	32.525±0.997	30.248±0.574	0.054	0.076	0.431	0.017*
MCV (fL)	52.096±0.316	52.056±0.415	50.74±0.397	0.015*	0.943	0.008**	0.025*
MCH (pg)	19.968±0.12	20.081±0.151	19.316±0.148	0.000**	0.590	0.001**	0.001**
MCHC (g/L)	383.357±0.814	385.875±0.995	380.8±1.238	0.009**	0.114	0.068	0.002**
RDW-CV (%)	14.954±0.152	14.556±0.177	15.044±0.19	0.181	0.137	0.698	0.075
RDW -SD (fL)	34.4±0.462	33.419±0.491	33.72±0.546	0.392	0.207	0.318	0.703
PLT (10^9/L)	455.571±22.536	356.063±25.348	313.28±23.89	0.000**	0.008**	0.000**	0.251
MPV (fL)	5.079±0.049	5.263±0.138	5.748±0.181	0.001**	0.353	0.000**	0.018*
PDW (fL)	14.625±0.035	14.881±0.08	15.116±0.142	0.001**	0.087	0.000**	0.124
PCT (%)	0.232±0.012	0.186±0.014	0.174±0.012	0.002**	0.019*	0.001**	0.513
TBIL (umol/L)	1.818±0.119	2.331±0.235	1.923±0.127	0.072	0.025*	0.591	0.077
DBIL (umol/L)	0.286±0.029	0.35±0.055	0.292±0.047	0.572	0.317	0.906	0.375
IDBIL (umol/L)	1.532±0.128	1.981±0.222	1.631±0.128	0.140	0.052	0.618	0.132
TP (g/L)	62.404±0.642	65.744±1.175	65.8±0.73	0.003**	0.007**	0.002**	0.963
ALB (g/L)	33.675±0.355	30.988±0.502	31.346±0.432	0.000**	0.000**	0.000**	0.581
GLO (g/L)	28.729±0.515	34.756±1.101	34.454±0.839	0.000**	0.000**	0.000**	0.801
A/G	1.186±0.023	0.9±0.034	0.931±0.032	0.000**	0.000**	0.000**	0.501
AST (U/L)	56.429±1.968	63.313±2.625	70.654±2.538	0.000**	0.059	0.000**	0.047*
ALT (U/L)	112.975±4.614	83.456±6.834	85.85±5.01	0.000**	0.000**	0.000**	0.769
ST/LT	0.516±0.023	0.822±0.062	0.868±0.042	0.000**	0.000**	0.000**	0.449
GGT (U/L)	6.571±0.274	5.75±0.512	10.654±1.124	0.000**	0.487	0.000**	0.000**
ALP (U/L)	212.393±14.908	111.438±9.854	125.038±8.302	0.000**	0.000**	0.000**	0.473
LDH (U/L)	603.458±46.008	629.667±87.027	609.348±66.661	0.962	0.786	0.945	0.834
PAB (mg/L)	13.536±1.425	13.525±1.848	20.392±8.168	0.572	0.999	0.339	0.411

ADA (U/L)	5.004±0.302	4.431±0.355	5.212±0.309	0.284	0.243	0.624	0.118
CHE (U/L)	1288.393±53.706	1005.188±55.89	1145.538±45.1	0.002**	0.001**	0.041*	0.084
5-NT (U/L)	2.179±0.272	1.994±0.301	1.954±0.226	0.797	0.648	0.524	0.923
AFU (U/L)	3.536±0.145	3.744±0.27	4.546±0.232	0.001**	0.513	0.000**	0.015*
TBA (umol/L)	41.457±5.794	28.419±6.929	39.827±5.376	0.327	0.154	0.836	0.217
BUN (mmol/L)	4.6±0.251	4.207±0.341	4.302±0.221	0.545	0.326	0.390	0.815
Cr (umol/L)	99.093±4.467	108.819±5.651	116.938±7.015	0.079	0.281	0.025*	0.375
UA (umol/L)	26.25±2.619	32.625±3.64	29.269±2.581	0.335	0.144	0.423	0.446
BMG (mg/L)	0.357±0.062	0.479±0.094	0.465±0.098	0.529	0.349	0.338	0.919
CYS-C (mg/L)	0.051±0.005	0.129±0.07	0.052±0.007	0.144	0.074	0.998	0.078
K (mmol/L)	5.087±0.085	4.763±0.2	4.975±0.086	0.171	0.061	0.452	0.223
Na (mmol/L)	126.411±0.437	125.85±0.679	124.804±0.511	0.068	0.480	0.022*	0.195
CL (mmol/L)	96.089±0.443	94.956±0.703	93.25±0.477	0.000**	0.151	0.000**	0.035*
TG (mmol/L)	1.544±0.092	1.911±0.097	2.098±0.097	0.000**	0.015	0.000**	0.215
CHOL (mmol/L)	5.594±0.183	4.841±0.28	5.728±0.358	0.115	0.086	0.723	0.047*
HDL (mmol/L)	3.769±0.076	2.945±0.111	3.019±0.096	0.000**	0.000**	0.000**	0.602
LDL (mmol/L)	3.312±0.137	2.846±0.223	3.463±0.258	0.161	0.149	0.586	0.060
APOA1 (g/L)	0.696±0.021	0.632±0.03	0.66±0.022	0.187	0.077	0.245	0.447
APOB (g/L)	0.02±0.002	0.032±0.005	0.033±0.003	0.008**	0.019*	0.004**	0.867
FMN (mmol/L)	1.46±0.039	1.446±0.044	1.447±0.039	0.961	0.821	0.804	0.991
GLU (mmol/L)	4.671±0.115	4.581±0.267	4.01±0.137	0.006**	0.707	0.002**	0.022*

Note: Most common used hematologic and biochemical parameters were tested. The marked * was significant difference (*P<0. 05*), and the marked ** was extremely significant difference (*P<0.01*). The unmarked letter indicated no statistical difference.

Supplementary Table 2. Comparison between the original data of transcriptional and the reference genome.

Sample	Total-reads	Total-map	Unique-map	Multi-map
M01	73761706	65238878(88.45%)	63195124(85.67%)	2043754(2.77%)
F01	88047074	78826105(89.53%)	76378332(86.75%)	2447773(2.78%)
M03	73432368	66498999(90.56%)	63964670(87.11%)	2534329(3.45%)
M07	70987850	64093554(90.29%)	62370381(87.86%)	1723173(2.43%)
F08	99443568	89609460(90.11%)	86429246(86.91%)	3180214(3.2%)
F12	81912444	73484901(89.71%)	70953267(86.62%)	2531634(3.09%)
M28	85232600	77444526(90.86%)	73071227(85.73%)	4373299(5.13%)
F28	84586998	75216823(88.92%)	71049159(84.0%)	4167664(4.93%)

Supplementary Table 3. Each profile significantly enriched in the KEGG pathway.

KEGG_Name	Corrected P-Value	Input	Profile
Adherens junction	0.041	MET|TCF7|LEF1|SMAD3	Profile 0
Gastric cancer	0.041	MET|TCF7|LEF1|SHC2|SMAD3	
Pathways in cancer	0.041	TCF7|PIM2|ESR2|SMAD3|MET|FOXO1|TRAF4|LEF1|FGFR3	
Hepatocellular carcinoma	0.041	MET|TCF7|LEF1|SHC2|SMAD3	
B cell receptor signaling pathway	0.0001	CR2|CD72|CD22|CD19|BLNK|CD79B|CD79A	Profile 1
Primary immunodeficiency	0.005	CD79A|BLNK|TNFRSF13C|CD19	
p53 signaling pathway	0.0007	GTSE1|CCNB1|CCNE1|RRM2	Profile 3
Cell cycle	0.003	CCNB1|MCM4|BUB1|CCNE1	
Oocyte meiosis	0.028	CCNB1|CCNE1|BUB1	
ECM-receptor interaction	0.002	ITGA2B|ITGB3|VWF|GP1BB	Profile 4
Hematopoietic cell lineage	0.002	EPOR|ITGA2B|ITGB3|GP1BB	
Platelet activation	0.004	ITGA2B|ITGB3|VWF|GP1BB	
Complement and coagulation cascades	0.013	VWF|C1QC|C1QA	
Focal adhesion	0.014	ITGA2B|ITGB3|MYL5|VWF	
Porphyrin and chlorophyll metabolism	0.024	BLVRB|ALAS2	
Prion diseases	0.029	C1QC|C1QA	
Human papillomavirus infection	0.042	ITGA2B|ITGB3|ISG15|VWF	
Staphylococcus aureus infection	0.042	C1QC|C1QA	
PI3K-Akt signaling pathway	0.049	EPOR|ITGA2B|ITGB3|VWF	
Cytokine-cytokine receptor interaction	0.006	LIF|CXCL2|CXCL8|BMPR1A|CXCL10|IL1R2|IL1R1	Profile 6
Amoebiasis	0.019	CXCL8|IL1R2|IL1R1|LAMA5	
Transcriptional misregulation in cancer	0.019	GADD45A|SPINT1|IL1R2|ETV7|CXCL8	

Supplementary Table 4. WGBS raw data quality control statistics.

Sample name	Raw_reads	clean_reads	Clean_ratio(%)	Q20(%)	Q30(%)	GC(%)	BS conversion rate(%)
F01	348184904	342475648	88.16	97.63	92.33	22.34	99.495
M01	346787724	341261170	89.03	97.62	92.28	22.18	99.596
M03	364891330	359118399	89.12	97.65	92.38	22.24	99.606
M07	356155312	350566471	88.84	97.49	92.00	22.40	99.611
F08	331811447	327684301	90.18	97.52	91.87	21.94	99.721
F12	325285795	321201535	88.65	97.82	92.83	22.24	99.542
M28	343875597	335720363	87.31	97.10	91.11	22.22	99.554
F28	347389962	339694259	88.15	96.92	90.66	22.00	99.672

Supplementary Table 5. The statistics of methylation status of c-site.

sample	C_covgMean	C(Mb)	CG(Mb)	CHG(Mb)	CHH(Mb)	MeanC(%)	MeanCG(%)	MeanCHG(%)	MeanCHH(%)
F01	10.4	9745.2	565.1	2070.9	7109.2	5.31	80.21	0.66	0.71
M01	11.2	10471.3	605.2	2224.8	7641.4	5.21	80.85	0.56	0.57
M03	11.6	10795.8	625.0	2299.3	7871.5	5.20	80.74	0.55	0.57
M07	11.5	10708.3	625.6	2285.0	7797.7	5.12	79.24	0.52	0.52
F08	11.4	10677.2	593.1	2244.6	7839.5	4.98	81.03	0.52	0.50
F12	10.3	9608.6	557.9	2043.8	7006.9	5.25	80.12	0.62	0.64
M28	10.4	9670.9	556.7	2059.7	7054.5	5.20	80.06	0.61	0.64
F28	11.1	10413.5	597.2	2210.9	7605.4	5.02	79.47	0.49	0.49
