# Supplementary material for: The immune and metabolic changes with age in giant panda blood by combined transcriptome and DNA methylation analysis
Source: Aging (Albany NY). 2020 Nov 7;12(21):21777–97. doi: 10.18632/aging.103990 (PMC11623972; doi:10.18632/aging.103990)
Supplement: Supplementary Table 6 [file aging-12-103990-s003.docx]

**Supplement Table 6. KEGG pathway significantly enriched by DMG in each group.**

| Group | KEGG pathway | pvalue | category | subcategories | DMGs |
| --- | --- | --- | --- | --- | --- |
| Young  vs.  Adult | Insulin secretion | 2.70E-03 | 5. Organismal Systems | 5.2 Endocrine system | *ADCY4、KCNMB2、CAMK2D、PRKCG、KCNN4、CREB3L1、PRKACB、RIMS2* |
|  | Aldosterone synthesis and secretion | 6.50E-03 | 5. Organismal Systems | 5.2 Endocrine system | *ADCY4、CACNA1H、CAMK2D、PRKCG、CACNA1G、CREB3L1、PRKACB* |
|  | Inflammatory mediator regulation of TRP channels | 7.40E-03 | 5. Organismal Systems | 5.7 Sensory system | *ADCY4、CAMK2D、ASIC4、PRKACB、NTRK1、PRKCG、MAPK10、IL1RAP* |
|  | Dilated cardiomyopathy | 1.10E-02 | 6. Human Diseases | 6.6 Cardiovascular disease | *ITGA8、ADCY4、ITGA1、SGCD、PRKACB、CACNA2D2、CACNG7* |
|  | Adrenergic signaling in cardiomyocytes | 1.20E-02 | 5. Organismal Systems | 5.3 Circulatory system | *ADCY4、SCN7A、CAMK2D、ADRA1A、CREB3L1、PRKACB、CACNA2D2、SCN5A、CACNG7* |
|  | Calcium signaling pathway | 4.40E-02 | 3. Environmental Information Processing | 3.2 Signal transduction | *ADCY4、ADRA1A、ERBB4、CACNA1H、CAMK2D、PRKCG、CACNA1G、PRKACB、EDNRA、HRC* |
|  | Vascular smooth muscle contraction | 4.50E-02 | 5. Organismal Systems | 5.3 Circulatory system | *ADCY4、ADRA1A、KCNMB2、PRKCG、PRKACB、EDNRA、CALD1* |
| Adult  vs.  Old | Small cell lung cancer | 0.0027 | 6. Human Diseases | 6.2 Cancer: specific types | *BCL2L1、TRAF1、LAMA5、CCND1、ITGA2、CHUK、RXRB、AKT2、LAMB3* |
|  | Glutamatergic synapse | 0.0045 | 5. Organismal Systems | 5.6 Nervous system | *ADCY4、PLCB1、GRIA1、PLCB4、ADCY2、GRIK2、SLC17A7、GRIN2B、TRPC1、SLC1A2、GRM3* |
|  | Dilated cardiomyopathy | 0.0076 | 6. Human Diseases | 6.6 Cardiovascular disease | *ITGA8、ADCY4、ITGB5、ADCY2、ITGA2、SGCD、CACNA2D2、CACNA2D3、LMNA* |
|  | Arrhythmogenic right ventricular cardiomyopathy (ARVC) | 0.0083 | 6. Human Diseases | 6.6 Cardiovascular disease | *ITGA8、TCF7、ITGB5、ITGA2、SGCD、CACNA2D2、CACNA2D3、LMNA* |
|  | AMPK signaling pathway | 0.012 | 3. Environmental Information Processing | 3.2 Signal transduction | *ADRA1A、ACACA、CCND1、PFKFB3、PPP2R2C、RPS6KB2、AKT2、CREB3L1、SREBF1、RAB14* |
|  | Acute myeloid leukemia | 0.013 | 6. Human Diseases | 6.2 Cancer: specific types | *RARA、TCF7、CCND1、CHUK、RPS6KB2、AKT2* |
|  | Adrenergic signaling in cardiomyocytes | 0.021 | 5. Organismal Systems | 5.3 Circulatory system | *ADCY4、ADRA1A、PLCB4、ADCY2、PLCB1、PIK3R5、PPP2R2C、KCNQ1、AKT2、CREB3L1、CACNA2D2、CACNA2D3* |
|  | Hippo signaling pathway | 0.023 | 3. Environmental Information Processing | 3.2 Signal transduction | *BMP7、TCF7、CCND1、BIRC5、CRB1、PPP2R2C、TEAD2、LIMD1、WWC1、WNT7B* |
|  | Sphingolipid signaling pathway | 0.024 | 3. Environmental Information Processing | 3.2 Signal transduction | *PLCB1、PLCB4、CERS3、S1PR2、PRKCE、S1PR5、AKT2、PPP2R2C、ASAH1* |
|  | Insulin secretion | 0.026 | 5. Organismal Systems | 5.2 Endocrine system | *ADCY4、PLCB1、KCNMB2、PLCB4、ADCY2、CREB3L1、KCNN1、RIMS2* |
|  | Focal adhesion | 0.028 | 4. Cellular Processes | 4.3 Cellular community - eukaryotes | *ITGA8、ITGB5、LAMA5、CCND1、ITGA2、AKT2、CAPN2、RASGRF1、LAMB3、PARVA、VAV3、PPP1R12A* |
|  | Chemokine signaling pathway | 0.03 | 5. Organismal Systems | 5.1 Immune system | *ADCY4、PLCB1、PIK3R5、PLCB4、ADCY2、GRK7、VAV3、CHUK、AKT2、ARRB1、ELMO1* |
|  | Cholinergic synapse | 0.031 | 5. Organismal Systems | 5.6 Nervous system | *ADCY4、PLCB1、PLCB4、ADCY2、PIK3R5、KCNQ1、AKT2、CREB3L1、SLC5A7* |
|  | Pathways in cancer | 0.039 | 6. Human Diseases | 6.1 Cancer: overview | *BCL2L1、PLCB1、BIRC5、AKT2、LAMB3、RARA、RPS6KB2、GLI1、JAG2、ITGA2、TRAF1、TCF7、PLCB4、LAMA5、CHUK、RXRB、FGFR3、ADCY4、ADCY2、CCND1、LPAR1、WNT7B* |
|  | cGMP-PKG signaling pathway | 0.043 | 3. Environmental Information Processing | 3.2 Signal transduction | *ADCY4、ADRA1A、KCNMB2、PLCB4、ADCY2、PLCB1、PRKCE、PIK3R5、PDE3B、AKT2、CREB3L1、PPP1R12A* |
|  | Aldosterone synthesis and secretion | 0.044 | 5. Organismal Systems | 5.2 Endocrine system | *ADCY4、PLCB1、PLCB4、ADCY2、CACNA1I、PRKCE、CACNA1G、CREB3L1* |
|  | Apoptosis | 0.049 | 4. Cellular Processes | 4.1 Transport and catabolism | *BCL2L1、TRAF1、BIRC5、PARP1、CHUK、CFLAR、AKT2、CAPN2、LMNA* |
|  | Thyroid hormone signaling pathway | 0.05 | 5. Organismal Systems | 5.2 Endocrine system | *PLCB1、PLCB4、CCND1、THRB、RXRB、MED12L、AKT2、PLCD1* |
| Young  vs.  Old | Neuroactive ligand-receptor interaction | 2.70E-04 | 3. Environmental Information Processing | 3.3 Signaling molecules and interaction | *AVPR1A、GHR、GRIA1、TSHR、GABRA1、GABRA2、S1PR3、S1PR2、PTGER2、PTGER3、HTR2B、EDN2、GRM3、ADRA1A、PTH2R、NPFFR1、MTNR1B、HTR1F、BDKRB1、GLRA3、CALCR、GRIK4、GRIN2B、LPAR3、AGTR1、LPAR1、LPAR6* |
|  | Adrenergic signaling in cardiomyocytes | 7.00E-03 | 5. Organismal Systems | 5.3 Circulatory system | *ADRA1A、SCN7A、SCN4B、PLCB4、ATP2A1、PLCB1、CAMK2D、ATP1B3、PPP2R2C、KCNQ1、AKT2、CREB3L1、PRKACB、AGTR1、ATP2B2* |
|  | Thyroid hormone synthesis | 7.90E-03 | 5. Organismal Systems | 5.2 Endocrine system | *LRP2、PLCB1、PLCB4、ATP1B3、PRKCG、CREB3L1、PRKACB、TSHR、PAX8* |
|  | Dopaminergic synapse | 9.00E-03 | 5. Organismal Systems | 5.6 Nervous system | *PLCB1、PLCB4、CAMK2D、GRIA1、PRKCG、GRIN2B、AKT2、CREB3L1、MAPK10、PPP2R2C、PRKACB、SLC18A2、SLC18A1* |
|  | Calcium signaling pathway | 9.50E-03 | 3. Environmental Information Processing | 3.2 Signal transduction | *BDKRB1、ADRA1A、AVPR1A、PLCB4、ATP2A1、PLCB1、CAMK2D、PLCZ1、CACNA1I、CACNA1E、PRKCG、PTGER3、PLCD1、PRKACB、HTR2B、AGTR1、ATP2B2* |
|  | Insulin secretion | 1.10E-02 | 5. Organismal Systems | 5.2 Endocrine system | *PLCB1、KCNMB2、PLCB4、CAMK2D、ATP1B3、PRKCG、KCNN4、CREB3L1、PRKACB、RIMS2* |
|  | Inflammatory mediator regulation of TRP channels | 1.20E-02 | 5. Organismal Systems | 5.7 Sensory system | *BDKRB1、PLCB1、PLCB4、CAMK2D、PRKACB、ASIC1、NTRK1、PRKCG、MAPK10、PTGER2、HTR2B* |
|  | Salivary secretion | 1.40E-02 | 5. Organismal Systems | 5.4 Digestive system | *ADRA1A、PLCB1、PLCB4、ATP1B3、PRKCG、GUCY1A3、KCNN4、PRKACB、ATP2B2* |
|  | Pathways in cancer | 1.50E-02 | 6. Human Diseases | 6.1 Cancer: overview | *BCL2L1、RET、AKT2、MAPK10、LAMB3、BAK1、FLT3、BCR、RARA、RPS6KB2、NTRK1、PTGER2、PTGER3、GLI1、TXNRD1、PLCB1、TCF7、PLCB4、CAMK2D、CHUK、PRKCG、COL4A4、APC2、FGFR2、PAX8、BDKRB1、BBC3、NOTCH3、PRKACB、LPAR3、AGTR1、LPAR1、LPAR6* |
|  | Pancreatic secretion | 1.50E-02 | 5. Organismal Systems | 5.4 Digestive system | *PLCB1、PLCB4、ATP2A1、ATP1B3、CPB1、KCNQ1、SLC4A4、TRPC1、PRKCG、ATP2B2* |
|  | cAMP signaling pathway | 2.00E-02 | 3. Environmental Information Processing | 3.2 Signal transduction | *GLI1、ATP2B2、ATP2A1、CAMK2D、VAV3、GRIA1、ATP1B3、PTGER2、PTGER3、GRIN2B、AKT2、CREB3L1、MAPK10、RRAS、PRKACB、TSHR、HTR1F、EDN2、PDE4D、PDE10A* |
|  | Amphetamine addiction | 2.30E-02 | 6. Human Diseases | 6.5 Substance dependence | *CAMK2D、GRIA1、PRKCG、GRIN2B、CREB3L1、PRKACB、SLC18A2、SLC18A1* |
|  | PI3K-Akt signaling pathway | 3.40E-02 | 3. Environmental Information Processing | 3.2 Signal transduction | *BCL2L1、ITGA8、GHR、ITGA1、EPHA2、FOXO3、AKT2、LAMB3、FLT3、FLT1、NTRK1、RPS6KB2、MAGI2、YWHAB、RELN、CHUK、PPP2R2C、COL4A4、PHLPP1、FGFR2、CREB3L1、LPAR3、VWF、LPAR1、LPAR6* |
|  | Amoebiasis | 3.50E-02 | 6. Human Diseases | 6.10 Infectious disease: parasitic | *RAB7B、PLCB4、PLCB1、MUC2、PRKCG、COL4A4、PRKACB、LAMB3* |
|  | Gastric acid secretion | 3.60E-02 | 5. Organismal Systems | 5.4 Digestive system | *PLCB1、PLCB4、CAMK2D、PRKACB、ATP1B3、PRKCG、KCNQ1、SLC26A7* |
