# Supplementary material for: The immune and metabolic changes with age in giant panda blood by combined transcriptome and DNA methylation analysis
Source: Aging (Albany NY). 2020 Nov 7;12(21):21777–97. doi: 10.18632/aging.103990 (PMC11623972; doi:10.18632/aging.103990)
Supplement: Supplementary Table 7 [file aging-12-103990-s004.pdf]

SUPPLEMENTARY TABLE

Supplementary Table 7. Differentially expressed and differentially methylated genes in each group.

Young vs. Adult	Adult vs. Old	Young vs. Old
COL24A1	*NME4*	*NCAPH*
SHISA8		*COL24A1*
MVB12B		*TEX30*
		ADAM28
		F13A1
		S100A6
		SOCS1
		TFPI2
		PPP1R14A
		ULK4
		ZNF704
		LPAR6
		WNK2
		KIF12
		PER1
		CD68
		PLA2G16
		RTN2
		KLHL14
		TCF7
		ZFHX2
		ISG15
		PROB1
		BACH2
		CREB3L1
		DNAH11
		VWF
		TNS2
		LOC100472228
		LOC100463922
		RALGPS2
		CUNH17orf99
		LOC100483371
		PDZD2
		TSGA10
		GAP43
