# Supplementary material for: The immune and metabolic changes with age in giant panda blood by combined transcriptome and DNA methylation analysis
Source: Aging (Albany NY). 2020 Nov 7;12(21):21777–97. doi: 10.18632/aging.103990 (PMC11623972; doi:10.18632/aging.103990)
Supplement: Supplementary Table 8 [file aging-12-103990-s005.docx]

**Supplementary Table 8. Basic information about giant pandas sampled in this experiment.**

| Name | | Gender | Age  (year) | Sampling year | Diet | Illness | Group |
| --- | --- | --- | --- | --- | --- | --- | --- |
| JB | female | | 1.5 | 2019 | milk, bamboo shoots, carrot, apple, liquid calcium |  | Young |
| BB | female | | 2 | 2019 | milk, steamde corn-bread, bamboo shoots, carrot, apple, bamboo, liquid calcium |  |  |
|  |  |  | 1.5 | 2018 | milk, bamboo shoots, carrot, apple, liquid calcium |  |  |
| BC | male | | 2 | 2019 | milk, steamde corn-bread, bamboo shoots, carrot, apple, bamboo, liquid calcium |  |  |
|  |  |  | 1.5 | 2018 | milk, bamboo shoots, carrot, apple, liquid calcium |  |  |
|  |  |  | 1.5 | 2018 | milk, bamboo shoots, carrot, apple, liquid calcium |  |  |
| FB |  | | 3 | 2019 | [steamed corn-bread, bamboo, carrot, apple, bamboo shoots, multivitamin formula with minerals](javascript:;) |  |  |
|  | male | | 2 | 2018 | milk, steamde corn-bread, bamboo shoots, carrot, apple, bamboo, liquid calcium |  |  |
|  |  | | 1.5 | 2017 | milk, bamboo shoots, carrot, apple, liquid calcium |  |  |
| JM | female | | 3 | 2019 | [steamed corn-bread, bamboo, carrot, apple, bamboo shoots, multivitamin formula with minerals](javascript:;) |  |  |
|  |  |  | 2 | 2018 | milk, steamde corn-bread, bamboo shoots, carrot, apple, bamboo, liquid calcium |  |  |
|  |  |  | 1.5 | 2017 | milk, bamboo shoots, carrot, apple, liquid calcium |  |  |
| MM | male | | 1.5 | 2019 | milk, bamboo shoots, carrot, apple, liquid calcium |  |  |
| NN | female | | 3 | 2018 | [steamed corn-bread, bamboo, carrot, apple, bamboo shoots, multivitamin formula with minerals](javascript:;) |  |  |
|  |  |  | 2 | 2017 | milk, steamde corn-bread, bamboo shoots, carrot, apple, bamboo, liquid calcium |  |  |
|  |  |  | 1.5 | 2018 | milk, bamboo shoots, carrot, apple, liquid calcium |  |  |
| PY | female | | 3 | 2018 | [steamed corn-bread, bamboo, carrot, apple, bamboo shoots, multivitamin formula with minerals](javascript:;) |  |  |
|  |  |  | 2 | 2017 | milk, steamde corn-bread, bamboo shoots, carrot, apple, bamboo, liquid calcium |  |  |
|  |  |  | 1.5 | 2018 | milk, bamboo shoots, carrot, apple, liquid calcium |  |  |
| QQ | male | | 3 | 2019 | [steamed corn-bread, bamboo, carrot, apple, bamboo shoots, multivitamin formula with minerals](javascript:;) |  |  |
|  |  |  | 2 | 2018 | milk, steamde corn-bread, bamboo shoots, carrot, apple, bamboo, liquid calcium |  |  |
| RY | male | | 2 | 2019 | milk, steamde corn-bread, bamboo shoots, carrot, apple, bamboo, liquid calcium |  |  |
|  |  |  | 1.5 | 2018 | milk, bamboo shoots, carrot, apple, liquid calcium |  |  |
| YX | male | | 3 | 2019 | [steamed corn-bread, bamboo, carrot, apple, bamboo shoots, multivitamin formula with minerals](javascript:;) |  |  |
|  |  |  | 2 | 2018 | milk, bamboo shoots, carrot, apple, liquid calcium |  |  |
| YH | female | | 3 | 2019 | steamed corn-bread, bamboo, carrot, apple, bamboo shoots, multivitamin formula with minerals |  |  |
|  |  |  | 2 | 2018 | milk, steamde corn-bread, bamboo shoots, carrot, apple, bamboo, liquid calcium |  |  |
|  |  |  | 1.5 | 2017 | milk, bamboo shoots, carrot, apple, liquid calcium |  |  |
| HH | male | | 7 | 2019 | steamed corn-bread, bamboo, carrot, apple, bamboo shoots, multivitamin formula with minerals |  | Adult |
| LL | male | | 9 | 2019 | [steamed corn-bread, bamboo, carrot, apple, bamboo shoots, multivitamin formula with minerals](javascript:;) |  |  |
| TS |  | | 13 | 2019 | [steamed corn-bread, bamboo, carrot, apple, bamboo shoots, multivitamin formula with minerals](javascript:;) |  |  |
|  | male | | 12 | 2018 | [steamed corn-bread, bamboo, carrot, apple, bamboo shoots, multivitamin formula with minerals](javascript:;) |  |  |
|  |  | | 11 | 2017 | [steamed corn-bread, bamboo, carrot, apple, bamboo shoots, multivitamin formula with minerals](javascript:;) |  |  |
| WW | male | | 13 | 2018 | steamed corn-bread, bamboo, carrot, apple, bamboo shoots, multivitamin formula with minerals |  |  |
|  |  |  | 12 | 2017 | steamed corn-bread, bamboo, carrot, apple, bamboo shoots, multivitamin formula with minerals |  |  |
| YM | female | | 15 | 2019 | steamed corn-bread, bamboo, carrot, apple, bamboo shoots, multivitamin formula with minerals |  |  |
|  |  |  | 14 | 2018 | steamed corn-bread, bamboo, carrot, apple, bamboo shoots, multivitamin formula with minerals |  |  |
| YY2 | male | | 16 | 2019 | [steamed corn-bread, bamboo, carrot, apple, bamboo shoots, multivitamin formula with minerals](javascript:;) |  |  |
|  |  |  | 15 | 2018 | [steamed corn-bread, bamboo, carrot, apple, bamboo shoots, multivitamin formula with minerals](javascript:;) |  |  |
| ZJ |  | | 18 | 2018 | steamed corn-bread, bamboo, carrot, apple, bamboo shoots, multivitamin formula with minerals |  |  |
|  | male | | 19 | 2019 | [steamed corn-bread, bamboo, carrot, apple, bamboo shoots, multivitamin formula with minerals](javascript:;) |  |  |
|  |  |  | 17 | 2017 | steamed corn-bread, bamboo, carrot, apple, bamboo shoots, multivitamin formula with minerals |  |  |
| DL |  | | 19 | 2017 | [steamed corn-bread, bamboo, carrot, apple, bamboo shoots, multivitamin formula with minerals](javascript:;) | amputation |  |
|  | male | | 20 | 2018 | steamed corn-bread, bamboo, carrot, apple, bamboo shoots, multivitamin formula with minerals | amputation | Old |
|  |  | | 21 | 2019 | steamed corn-bread, bamboo, carrot, apple, bamboo shoots, multivitamin formula with minerals | amputation |  |
| YP | female | | 23 | 2019 | steamed corn-bread, bamboo, carrot, apple, bamboo shoots, multivitamin formula with minerals | hypertension |  |
|  |  |  | 22 | 2018 | steamed corn-bread, bamboo, carrot, apple, bamboo shoots, multivitamin formula with minerals | hypertension |  |
|  |  |  | 21 | 2017 | [steamed corn-bread, bamboo, carrot, apple, bamboo shoots, multivitamin formula with minerals](javascript:;) | hypertension |  |
| YY | female | | 27 | 2018 | steamed corn-bread, bamboo, carrot, apple, bamboo shoots, multivitamin formula with minerals |  |  |
|  |  |  | 28 | 2019 | steamed corn-bread, bamboo, carrot, apple, bamboo shoots, multivitamin formula with minerals |  |  |
| YY3 |  | | 27 | 2019 | [steamed corn-bread, bamboo, carrot, apple, bamboo shoots, multivitamin formula with minerals](javascript:;) | hypertension |  |
|  | female | | 26 | 2018 | [steamed corn-bread, bamboo, carrot, apple, bamboo shoots, multivitamin formula with minerals](javascript:;) | hypertension |  |
|  |  | | 25 | 2017 | steamed corn-bread, bamboo, carrot, apple, bamboo shoots, multivitamin formula with minerals | hypertension |  |
| ZX | female | | 22 | 2018 | [steamed corn-bread, bamboo, carrot, apple, bamboo shoots, multivitamin formula with minerals](javascript:;) | cataract, hypertension |  |
| HZ | female | | 23 | 2018 | steamed corn-bread, bamboo, carrot, apple, bamboo shoots, multivitamin formula with minerals |  |  |
|  |  |  | 22 | 2017 | steamed corn-bread, bamboo, carrot, apple, bamboo shoots, multivitamin formula with minerals |  |  |
| QY | female | | 27 | 2018 | [steamed corn-bread, bamboo, carrot, apple, bamboo shoots, multivitamin formula with minerals](javascript:;) |  |  |
|  |  |  | 26 | 2017 | steamed corn-bread, bamboo, carrot, apple, bamboo shoots, multivitamin formula with minerals |  |  |
| SL |  | | 23 | 2019 | [steamed corn-bread, bamboo, carrot, apple, bamboo shoots, multivitamin formula with minerals](javascript:;) | hypertension |  |
|  | female | | 21 | 2017 | steamed corn-bread, bamboo, carrot, apple, bamboo shoots, multivitamin formula with minerals | hypertension |  |
|  |  | | 22 | 2018 | steamed corn-bread, bamboo, carrot, apple, bamboo shoots, multivitamin formula with minerals | hypertension |  |
| XM |  | | 27 | 2018 | [steamed corn-bread, bamboo, carrot, apple, bamboo shoots, multivitamin formula with minerals](javascript:;) |  |  |
|  | male | | 28 | 2019 | steamed corn-bread, bamboo, carrot, apple, bamboo shoots, multivitamin formula with minerals |  |  |
|  |  | | 26 | 2017 | steamed corn-bread, bamboo, carrot, apple, bamboo shoots, multivitamin formula with minerals |  |  |
| DD | male | | 24 | 2019 | [steamed corn-bread, bamboo, carrot, apple, bamboo shoots, multivitamin formula with minerals](javascript:;) | hypertension |  |
|  |  |  | 23 | 2018 | [steamed corn-bread, bamboo, carrot, apple, bamboo shoots, multivitamin formula with minerals](javascript:;) | hypertension |  |
|  |  |  | 22 | 2017 | [steamed corn-bread, bamboo, carrot, apple, bamboo shoots, multivitamin formula with minerals](javascript:;) | hypertension |  |
| FF | female | | 22 | 2018 | [steamed corn-bread, bamboo, carrot, apple, bamboo shoots, multivitamin formula with minerals](javascript:;) |  |  |
|  |  |  | 21 | 2017 | [steamed corn-bread, bamboo, carrot, apple, bamboo shoots, multivitamin formula with minerals](javascript:;) |  |  |
